# Protective effect and mechanism of Lactobacillus on cerebral ischemia reperfusion injury in rats

**DOI:** 10.1590/1414-431X20187172

**Published:** 2018-05-17

**Authors:** Shi Wanchao, Ma Chen, Su Zhiguo, Xie Futang, Shi Mengmeng

**Affiliations:** Cerebrovascular Disease Treatment Center, No.5 Tianjin Center Hospital, Tianjin, China

**Keywords:** Cerebral ischemia reperfusion injury, Inactivated Lactobacillus, Apoptosis, NF-kappa B, TLR-4

## Abstract

The present study was designed to investigate the protective effects and mechanism of inactivated lactobacillus (ILA) on cerebral ischemia reperfusion injury (CIRI) in rats. In this experiment, 30 male Sprague Dawley rats were randomly divided into control group, IRI groups, and ILA group. A middle cerebral artery occlusion and reperfusion model was prepared. The rats were killed after 24 hours of recovery of blood flow of cerebral ischemia resulting from 60-min occlusion. The cerebral infarction volume and neurological scores were assayed by staining and behavioral observation. Malondialdehyde (MDA) and superoxide dismutase (SOD) levels were assayed by biochemical kits. Cell apoptosis was assayed by Tunnel and the Toll-like receptor (TLR)-4, IkB, and A20 were assayed by western blot. The neurobehavioral scores in IRI rats were significantly lower compared to the control group while ILA improved the neurobehavioral scores of the ILA groups. The cerebral infarction volume and neural cell apoptosis of rats in the ILA groups decreased significantly compared with those in the IRI group. In addition, MDA level in the ILA groups decreased whereas SOD activity increased compared to the IRI group. Moreover, ILA also inhibited the expression of TLR-4 and promoted the expression of IkB and A20. ILA inhibited the apoptosis of neural cells, decreased cerebral infarction volume, and reduced oxidative stress through inhibition of TLR-4/NF-kappa B signaling, improving neurobehavioral scores. Thus from the present study it was concluded that ILA has protective effect on CIRI.

## Introduction

Ischemia is a restriction in blood supply to tissues, causing a shortage of oxygen and glucose needed for cellular metabolism. Reperfusion is the restoration of blood flow to an organ or tissue after having been blocked. Cerebral ischemia reperfusion injury (CIRI) is ischemia reperfusion injury that is occurring in the brain. There are various disorders characterized by IRI, among which myocardial infarction and stroke are leading causes of mortality and disability in the world (1). The pathophysiological mechanism of CIRI includes generation of reactive oxygen species (ROS) and apoptosis of neural cells (2). Reperfusion of ischemic tissues is often associated with microvascular injury where more ROS are generated following reperfusion. Similarly, after CIRI, there is excessive production of ROS in the cerebrum because of the imbalance between oxidation and antioxidant system. The radicals are very active with other molecules such as DNA and lipids. Mitochondria are the main sites of ROS production and the main targets of ROS after cerebral ischemia leading to injuries of phospholipid membrane and abnormality in oxidative phosphorylation process of ATP (3).

Although oxygen levels are restored upon reperfusion, a surge in the generation of ROS occurs and pro-inflammatory neutrophils infiltrate ischemic tissues to exacerbate ischemic injury. The restored blood flow reintroduces oxygen within cells, which exacerbates ischemic injury and damages cellular proteins, DNA, and the plasma membrane, leading to cell apoptosis. The inflammation-mediated mechanisms where the inflammatory mediators play a crucial role are mainly involved in CIRI. In a recent study, an involvement of Toll-like receptors (TLRs) was found in the induction of inflammatory responses, with an increase in TLR-4 after CIRI. Moreover, the up-regulation of NF-κB signaling pathway after CIRI increases the inflammatory process (4) whereas downregulation of NF-κB signaling pathway alleviates the inflammation and CIRI (5). Therefore, TLR-4/NF-κB signaling is recognized as a good target for management of CIRI.

The human body is colonized by a vast number of microbes collectively referred to as the human microbiota, which reside on or within human tissues and biofluids. The microbiota have been found to be crucial for immunologic, hormonal, and metabolic homeostasis of their host. The link between these microbes and our health is the focus of a growing number of research initiatives and new insights are emerging rapidly (6). Recently, various studies have been done on microbe products because of their obvious effects in inflammation and immunity regulation to develop safe and effective agents for treatment of CIRI (7). *Lactobacillus* is a genus of Gram-positive, facultative anaerobic or micro-aerophilic, rod-shaped, non-spore-forming bacteria. In humans, they constitute a significant component of the microbiota at a number of body sites, such as the digestive system, urinary system, and genital system. Recent studies showed that Lactobacillus could provide anti-inflammatory effects in myocardial infarction through activating Treg immune cells (8). In addition, soluble factors from *Lactobacillus reuteri* CRL1098 inhibited endotoxin-induced acute lung injury through NF-kB and PI3K inhibition (9). ILA paracasei down-regulated the LPS-induced production of IL-1, TNF-α, and IL-6 through induction of negative regulators of the NF-κB signaling pathway in a TLR2-IRAK4-dependent manner (10). Moreover, novel findings suggested that *Lactobacillus paracasei* subsp. paracasei NTU 101-fermented products had neuroprotective effects in the brain and attenuated hypertension-induced vascular dementia (11). However, the neuroprotective effect of lactobacillus on CIRI is not clearly understood and the ability of lactobacillus to antagonize the inflammatory signaling pathway in brain remains to be determined.

Hence, presently it is known that ILA inhibits the apoptosis of neural cells and reduces oxidative stress through down-regulation of TLR4/NF-κB signaling pathway. However, we further sought to explore the potential role and mechanism of ILA in CIRI.

## Material and Methods

### Animals

Male Sprague Dawley rats (30, weight, 250∼300 g) were obtained from the Center of Experimental Animals, Wuhan University, Hubei, China. The animals were raised in a 12-h dark-light cycle with food and water available. All experimental procedures involving animals were in accordance with the Guide for the Care and Use of Laboratory Animals by National Institutes of Health and the protocol was approved by the Animal Experimentation Ethics Committee of Wuhan University.

### Preparation of middle cerebral artery occlusion and reperfusion model

The rats were anesthetized with pentobarbital (12.5 mg/kg) and maintained with α-chloralose (75 mg/kg) during surgery and preparation. Rats were placed on an operating table in a supine position under an operating microscope. The right side of common carotid artery (CCA), external carotid artery (ECA), and internal carotid artery (ICA) were exposed with blunt dissection. Then, the first branch of ECA was ligated at 0.2 cm away from the heart end and cut off, ensuring a residual length not less than 0.5 cm. Next, the traffic between ECA and ICA was ligated and cut off and the blood flow of CCA and ICA was temporarily blocked with an artery clamp. Then, a longitudinal small incision at ECA stump was done and a nylon thread about 0.2 mm in diameter with a line bolt about 0.25 mm was inserted from the right side of the ECA residual end into the ICA. The thread was slowly inserted to reach the terminal ICA until a mild resistance was felt and the ligature on the ICA was tightened. Then, the incision was temporarily closed and the rat was placed back in the cage. One hour after the occlusion, the rat was re-anesthetized and the thread was gently removed. After recovery from anesthesia, the rat was returned to the cage. Animals in the control group underwent the same operations without the ligation of ICA (12).

### Reagents and treatments

ILA amylovorus DSM 16698T (ILA) was obtained from the Department of Microbiology, No. 5 Tianjin Center Hospital (China). The rats were given intravenously three different 0.1-mL doses of ILA (10^6^, 10^7^ and 10^8^ cfu/mL). Rats were randomly assigned into five groups (6 in each group): sham operation group, IRI group, IRI+ ILA (10^6^ cfu/mL) group, IRI+ ILA (10^7^ cfu/mL) group, and IRI+ ILA (10^8^ cfu/mL). ILA solution was administered via intravenous injection 2 h before CIRI.

### Evaluation of neurological scores

Neurological scores were evaluated by an investigator blinded to the experimental groups after 24-h reperfusion. The scoring system was based on a 5-point scale system described previously (13): 0, no neurological deficit; 1, failure to extend contralateral forelimb fully; 2, rotate to the opposite side under slight stimulation; 3, falling to the left; 4, inability to walk spontaneously and decreased levels of consciousness. The animals were killed by pentobarbital sodium injection (100 mg/kg, *iv*) after the procedure.

### Cerebral infarction size

2,3,5-triphenyltetrazolium chloride (TTC) staining was used to assess the cerebral infarction size. Following cerebral ischemia/reperfusion injury for 24 hours, rats were euthanized under deep anesthesia with pentobarbital sodium (i.v. 100 mg/kg). The brains were quickly removed and frozen at -20°C for 10 min. Then, sections with a 2-mm thickness were cut using brain microtome and the slices were stained with 2% TTC solution (Sigma, USA) for 20 min at 37°C in a dark place followed by fixation with 4% paraformaldehyde overnight. The normal brain tissue was stained dark red, whereas the infracted area remained pale white. The infarct area was analyzed by an observer blinded to the experimental conditions using Image-Pro plus 6 and the percentage of infarction volume was calculated as the ratio of the infarcted area to the overall area.

### Determination of SOD and MDA

After obtaining the whole brain, specimens were cut in the middle from the left frontal pole to occipital lobe. The tissues from the occipital lobe were weighed and homogenized. The protein concentration was determined by BCA assay kit (Beyotime Biotech, China). The superoxide dismutase (SOD) activities were evaluated by hydroxylamine method while malondialdehyde (MDA) contents were evaluated by glucosinolates barbituric acid method, according to kit instructions (Jiancheng, China).

### TUNEL assay to determine neural cell apoptosis

The formalin-fixed frontal cortex tissues were embedded in paraffin and sectioned at 4-μm thickness; 5 pieces for each tissue were cut with a microtome. The sections were analyzed by the TUNEL assay to detect the apoptotic cells. The TUNEL assay kit was purchased from Roche Molecular Biochemicals, Mannheim, Germany, and the experiment was conducted according to manufacturer's protocol. Apoptotic cells with condensed nuclei were stained brown, while normal cells were large, round, and not stained. The positive cells were analyzed under a light microscope by an investigator blinded to the experimental groups. The extent of brain injury was evaluated by the percentage of TUNEL-positive cells.

### Western blot analysis

The other half of frontal cortex tissues was used for protein extraction. Total proteins were extracted from the infarct cortex and protein concentrations were determined using a BCA assay kit as instructed by the manufacturer (Beyotime Biotech, China). Samples containing 80 µg proteins were loaded and separated by electrophoresis on 12% SDS-polyacrylamide gels. Subsequently, proteins were transferred onto PVDF membranes, blocked for two hours with 5% nonfat dry milk at room temperature, and incubated with rabbit anti-TLR4 (1 : 300, Abcam, USA), anti- IκB (1 : 2000, Proteintech, USA), anti-A20 (1 : 2000, Proteintech), anti-GAPDH (1 : 200, Boster, China) at 4°C on a shaker overnight and then washed followed by incubation with the corresponding HRP-conjugated secondary antibody (Boster). Finally, ECL solution was added to reveal the bands and the gray value was analyzed by ImageJ software.

### Statistical analysis

Data are reported as means±SD. Differences between the mean values were evaluated by one-way ANOVA, followed by Duncan's multiple range test. For all tests, values of P<0.05 were considered to be statistically significant. Statistical analyses were performed using SPSS 17.0 software.

## Results

### Effect of ILA on neuroprotection

**Figure 1.**
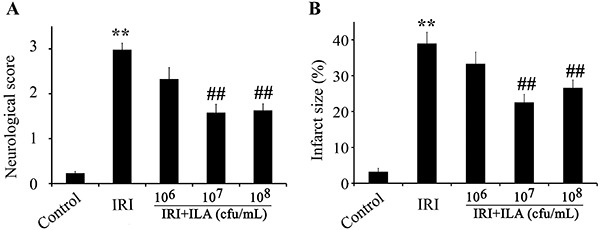
Effect of different concentrations of inactivated lactobacillus (ILA) on neurological deficit (*A*) and infarct size (*B*) after cerebral ischemia reperfusion injury (IRI) (n=4). Data are reported as means±SD. **P<0.01 *vs* control; ^##^P<0.01 *vs* IRI (ANOVA, followed by Duncan's multiple range test).

To determine the neuroprotective effects of ILA against CIRI, an IRI model was established and the neurological scores and cerebral infarct sizes 24 hours after injury were assessed. The results showed that pretreatment of 100 μL ILA intravenous injection (10^7^ and 10^8^ cfu/mL) significantly improved the neurologic scores (F=12.93 and 12.77, respectively, both P<0.01, Figure 1A). As demonstrated by the TTC-stained sections, the infarct sizes were also reduced at the same doses compared with IRI group (F=16.58 and 14.97, respectively, both P<0.01, Figure 1 B).

### Effect of ILA on SOD and MDA

**Figure 2.**
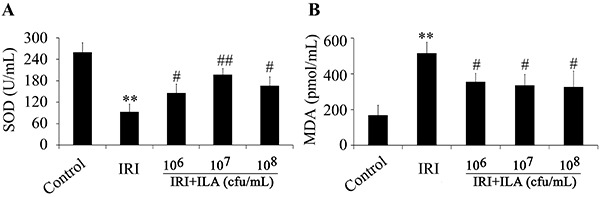
Effect of different concentrations of inactivated lactobacillus (ILA) on the levels of superoxide dismutase (SOD) (*A*) and malondialdehyde (MDA) (*B*) in occipital lobe tissues after ischemia reperfusion injury (IRI) (n=4). Data are reported as means±SD. **P<0.01 *vs* Control; ^##^P<0.01 and ^#^P<0.05 *vs* IRI (ANOVA, followed by Duncan's multiple range test).

To identify the downstream effects in the TLR4/NF-κB signaling pathway, the levels of SOD and MDA in occipital lobe tissues were examined by biochemical methods (Figure 2). The results demonstrated that SOD activity (F=14.65, P<0.01) in brain decreased and MDA content (F=15.33, P<0.01) increased in the IRI group while in ILA groups, SOD (Figure 2A) activities increased (F=7.66, 11.54, and 9.43, respectively), and MDA contents (F=8.68, 9.54, and 9.87, respectively, all P<0.05) decreased (Figure 2B). These findings suggested that ILA suppressed brain oxidative stress after ischemia.

### Effect of ILA on neural cell apoptosis after CIRI

**Figure 3.**
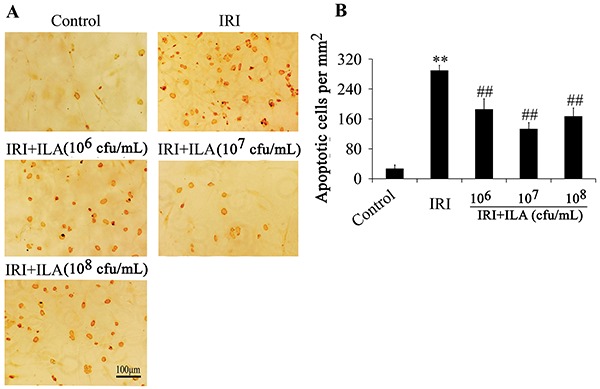
Effect of different concentrations of inactivated lactobacillus (ILA) on ischemia reperfusion injury (IRI) in neural cell apoptosis in frontal cortex was assayed by TUNEL assay (*A*) (n=4). Scale bar: 100 μM. *B*, Data are reported as means±SD. **P<0.01 *vs* control; ^##^P<0.01 *vs* IRI (ANOVA, followed by Duncan's multiple range test).

As shown in Figure 3, we found that the apoptosis of neural cells in IRI group compared with the control group was significantly increased (F=29.45, P<0.01) while in ILA groups, it was decreased (F=16.52, 14.39, and 15.88, respectively, all P<0.01), suggesting the antiapoptotic activity of ILA in CIRI.

### Effect of ILA on the TLR4/NF-κB signaling pathway in IRI animals

**Figure 4.**
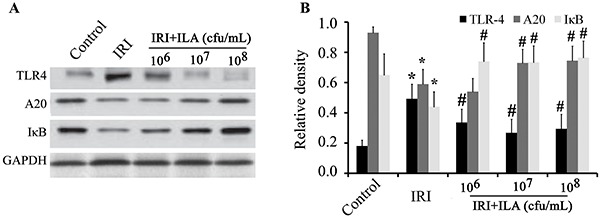
Effect of different concentrations of inactivated lactobacillus (ILA) after ischemia reperfusion injury (IRI) on the protein levels of TLR4, A20, and I-κB assayed by western blot analysis (n=4). Data are reported as means±SD. *P<0.05 *vs* control; ^#^P<0.05 *vs* IRI (ANOVA, followed by Duncan's multiple range test).

The protein levels of TLR4 (F=10.58, P<0.05) were increased and I-κB and A20 (F=11.87 and 9.69, respectively, both P<0.05) were decreased in the IRI group. TLR4 was decreased (F=9.45, 11.33, and 10.67, respectively, all P<0.05), and I-κB (F=8.94 and 9.03, respectively, both P<0.05) and A20 (F=12.57, 12.63, and 12.66, respectively, all P<0.05) were increased in the ILA groups compared with the IRI group (P<0.05) (Figure 4). These results suggested that the TLR4/NF-κB signaling pathway was involved with the protective effects of ILA on CIRI.

## Discussion

Ischemic cerebral vascular disease (ICVD), a disorder resulting from inadequate blood flow in the vessels that supply the brain, is a highly disabling and deadly disease, with a mortality rate greater in men than in women. In the treatment of ICVD, the reconstruction of blood flow or enhancement of the blood supply in the ischemic region is essential for the repair of ischemic brain tissue, but it also poses a problem of reperfusion injury. Other available therapeutic approaches for ICVD, such as thrombolytic tissue plasminogen activator, have a limited therapeutic window. Therefore, it is essential that new therapies be developed to improve ICVD outcome. Recent studies have elucidated the mechanisms of CIRI at cellular and molecular levels, including the generation of large amounts of oxygen free radicals, immune inflammatory damage, and apoptosis, (14) helping the development of new therapies for CIRI.

In recent years, it was shown that ILA have protective effects in inflammatory diseases and neuropathology (15). To evaluate the success of model establishment, neurological scores are assessed because there is a positive correlation between neurological scores, infarct volume size, and cerebral blood flow changes. In the present study, we found that ILA significantly improved the neurologic scores and the infarct sizes. Moreover, different concentrations of ILA can protect against CIRI, however 10^7^ cfu/mL of ILA showed better protection.

Reactive oxygen species are produced from mitochondrial energy metabolism via oxidative phosphorylation and are very harmful to the body. Therefore, their neutralization or clearance from our body is maintained by antioxidant enzymes, such as glutathione, superoxide dismutase, and catalase. Following ischemia, the level of various reactive oxygen species from different sources is significantly increased resulting in more use of endogenous antioxidant compounds as a compensation mechanism. When the injury is recurrent or prolonged, compensatory responses fail to maintain normal physiological oxidative state, resulting in oxidative stress with activation of subsequent signaling events leading to inflammatory responses and tissue damage. Reperfusion at ischemic areas enhances ischemic damage. When CIRI occurs, it results in the accumulation of ROS such as superoxide anions, hydroxyl radicals, hypochlorous acid, hydrogen peroxide, and nitric oxide-derived peroxynitrite through various ways. This induces lipid peroxidation leading to a series of pathophysiological changes such as damage to the structure of the biofilm, and malfunction of ion transport, biomass production and organelles, thereby aggravating IRI (16). MDA, as the biological product of ROS and lipid peroxidation, reflects the degree of lipid peroxidation in the body and thus indirectly reflect the degree of IRI (17). SOD is the main antioxidant enzyme related to the ability to remove free radicals (18). Thus, an imbalance between excess production of free radicals during IRI and inability of the body to counteract or detoxify their harmful effects through neutralization by antioxidants (SOD) results in oxidative stress. This study found that ILA increased SOD activity and decreased MDA content in the brain, further confirming the CIRI protection of ILA by inhibiting oxidative stress.

Apoptosis is a gene-controlled autonomous cell death process, also known as programmed cell death under physiological conditions. It is one of the mechanisms to maintain the stability of the internal environment. In recent years, apoptosis has been found to be related to some pathological conditions. Excessive inhibition or over enhancement can lead to the development of disease. Evidence shows that neuronal apoptosis is the basic form of delayed neuronal death after cerebral ischemia and reperfusion (19). It is likely that apoptotic neurons are responsible for successive injury. By salvaging these apoptotic neurons, the pathological outcome could be improved. In the present study, we found that apoptotic neural cells in the frontal cortex were significantly increased in the IRI group and ILA significantly inhibited the apoptosis of neural cells, suggesting the antiapoptotic activity of ILA in CIRI.

TLRs is a family of transmembrane pattern-recognition receptors, which can be activated by endogenous damage-associated molecular patterns released from injured or stressed cells under ischemic situation. TLRs represent a key molecular link between tissue injury, infection, and inflammation. The activation of TLR signaling pathway occurs by binding of their ligands and results in NF-κB activation, which in turn acts as a direct or indirect transcriptional activator of pro-inflammatory cytokine and chemokine (IL-1α/β, IL-6, IL-8, MIP-1α/β, TNF-α) gene expression. TLR4/NF-κB has been used as a therapeutic target for CIRI, because the overexpression of various inflammatory media including TNF-α, IL-1β, and IL-6 and the degeneration and apoptosis of neural cells were induced after TLR4/NF-κB activation (20). Recent studies have also shown that TLR4 is highly induced after CIRI and CIRI is alleviated in TLR4-deficient mice (21). In the present study, we evaluated the effects of ILA on the expression of TLR4, IκB, and NF-κB inhibitor A20, which indicated that the underlying mechanism of the neuroprotective effects involves the inhibition of the TLR4/NF-κB signaling pathway. We found that TLR4 was decreased and IκB and A20 were increased in the ILA groups compared with the IRI group.

In summary, the present study demonstrated that ILA alleviated CIRI by reducing oxidative stress and apoptosis of neural cells through inhibition of TLR4/NF-κB-mediated inflammatory signaling pathway.

## References

[1] Kalogeris T, Baines CP, Krenz M, Korthuis RJ (2016). Ischemia/Reperfusion. Compr Physiol.

[2] Zhang B, Yang N, Mo ZM, Lin SP, Zhang F (2017). IL-17A Enhances Microglial Response to OGD by Regulating p53 and
PI3K/Akt Pathways with Involvement of ROS/HMGB1. Front Mol Neurosci.

[3] Sun J, Li YZ, Ding YH, Wang J, Geng J, Yang H (2014). Neuroprotective effects of gallic acid against
hypoxia/reoxygenation-induced mitochondrial dysfunctions in vitro and
cerebral ischemia/reperfusion injury in vivo. Brain Res.

[4] Wu L, Tan JL, Wang ZH, Chen YX, Gao L, Liu JL (2015). ROS generated during early reperfusion contribute to intermittent
hypobaric hypoxia-afforded cardioprotection against postischemia-induced
Ca(2+) overload and contractile dysfunction via the JAK2/STAT3
pathway. J Mol Cell Cardiol.

[5] Kim E, Kim HC, Lee S, Ryu HG, Park YH, Kim JH (2017). Dexmedetomidine confers neuroprotection against transient global
cerebral ischemia/reperfusion injury in rats by inhibiting inflammation
through inactivation of the TLR-4/NF-κB pathway. Neurosci Lett.

[6] Li L, Liu M, Kang L, Li Y, Dai Z, Wang B (2016). HHEX: A Crosstalker between HCMV Infection and Proliferation of
VSMCs. Front Cell Infect Microbiol.

[7] Kim MS, Lee S, Jung N, Lee K, Choi J, Kim SH (2017). The vitamin D analogue paricalcitol attenuates hepatic
ischemia/reperfusion injury through down-regulation of Toll-like receptor 4
signaling in rats. Arch Med Sci.

[8] Wang L, Wu G, Qin X, Ma Q, Zhou Y, Liu S (2015). Expression of Nodal on Bronchial Epithelial Cells Influenced by
Lung Microbes Through DNA Methylation Modulates the Differentiation of
T-Helper Cells. Cell Physiol Biochem.

[9] Danilo CA, Constantopoulos E, McKee LA, Chen H, Regan JA, Lipovka Y (2017). Bifidobacterium animalis subsp. lactis 420 mitigates the
pathological impact of myocardial infarction in the mouse. Benef Microbes.

[10] Griet M, Zelaya H, Mateos MV, Salva S, Juarez GE, de Valdez GF (2014). Soluble factors from Lactobacillus reuteri CRL1098 have
anti-inflammatory effects in acute lung injury induced by lipopolysaccharide
in mice. PLoS One.

[11] Sun KY, Xu DH, Xie C, Plummer S, Tang J, Yang XF (2017). Lactobacillus paracasei modulates LPS-induced inflammatory
cytokine release by monocyte-macrophages via the up-regulation of negative
regulators of NF-κB signaling in a TLR2-dependent manner. Cytokine.

[12] Ratilal BO, Arroja MM, Rocha JP, Fernandes AM, Barateiro AP, Brites DM (2014). Neuroprotective effects of erythropoietin pretreatment in a
rodent model of transient middle cerebral artery occlusion. J Neurosurg.

[13] Cheng MC, Pan TM (2017). Prevention of hypertension-induced vascular dementia by
lactobacillus paracasei subsp. paracasei NTU 101-fermented
products. Pharm Biol.

[14] Shen J, Zhu Y, Huang K, Jiang H, Shi C, Xiong X (2016). Buyang Huanwu Decoction attenuates H2O2-induced apoptosis by
inhibiting reactive oxygen species-mediated mitochondrial dysfunction
pathway in human umbilical vein endothelial cells. BMC Complement Altern Med.

[15] Bond DM, Morris JM, Nassar N (2017). Study protocol: evaluation of the probiotic Lactobacillus
Fermentum CECT5716 for the prevention of mastitis in breastfeeding women: a
randomised controlled trial. BMC Pregnancy Childbirth.

[16] Liu CW, Yang F, Cheng SZ, Liu Y, Wan LH, Cong HL (2017). Rosuvastatin postconditioning protects isolated hearts against
ischemia-reperfusion injury: The role of radical oxygen species,
PI3K-Akt-GSK-3β pathway, and mitochondrial permeability transition
pore. Cardiovasc Ther.

[17] Wang Z, Yu J, Wu J, Qi F, Wang H, Wang Z (2016). Scutellarin protects cardiomyocyte ischemia-reperfusion injury by
reducing apoptosis and oxidative stress. Life Sci.

[18] Wu SZ, Tao LY, Wang JN, Xu ZQ, Wang J, Xue YJ (2017). Amifostine pretreatment attenuates myocardial
ischemia/reperfusion injury by inhibiting apoptosis and oxidative
stress. Oxid Med Cell Longev.

[19] Wang Z, Bu J, Yao X, Liu C, Shen H, Li X (2017). Phosphorylation at S153 as a functional switch of
phosphatidylethanolamine binding protein 1 in cerebral ischemia-reperfusion
injury in rats. Front Mol Neurosci.

[20] Zhang X, Du Q, Yang Y, Wang J, Dou S, Liu C (2017). The protective effect of Luteolin on myocardial
ischemia/reperfusion (I/R) injury through TLR4/NF-κB/NLRP3 inflammasome
pathway. Biomed Pharmacother.

[21] Hua F, Ha T, Ma J, Li Y, Kelley J, Gao X (2007). Protection against myocardial ischemia/reperfusion injury in
TLR4-deficient mice is mediated through a phosphoinositide
3-kinase-dependent mechanism. J Immunol.

